# Balancing performance-based expectations with a holistic perspective on coaching: a qualitative study of Swedish women’s national football team coaches’ practice experiences

**DOI:** 10.1080/17482631.2017.1358580

**Published:** 2017-08-16

**Authors:** Eva-Carin Lindgren, Natalie Barker-Ruchti

**Affiliations:** ^a^ School of Health and Welfare, Halmstad University, Halmstad, Sweden; ^b^ Department of Food and Nutrition, and Sport Science, University of Gothenburg, Gothenburg, Sweden

**Keywords:** Caring, coaching, holistic perspective, football, national team, women

## Abstract

**Purpose**: The purpose of this study was to explore how an exclusive sample of women’s national football team coaches described how they implement careful coaching while facing social and organizational pressure to win medals. **Method**: To consider coaches’ negotiations, we drew on Noddings’ concept of caring. Using an interpretive research paradigm, we conducted in-depth interviews with five Swedish women’s national football team coaches. An abductive approach was used to simultaneously process the theoretical framework of “ethics of care” and the empirical data. **Results**: The coaches unanimously adopted a holistic perspective to coaching. The coaching strategies they described included promoting players’ development, well-being, and sustainable elite performance; listening to the players’ voices and engaging in dialogue; and creating a positive environment and promoting fair play. **Conclusions**: These findings demonstrate that the women coaches, despite performance pressure, adopt caring coaching in the form of Noddings’ pedagogical modelling, dialogue, and confirmation strategies, and provide an example of how coaches can adopt caring, holistic, and athlete-centred coaching while working at the highest level of competitive sport and achieving competitive success.

## Introduction

Coaches at the upper echelons of sport are responsible for serving the interests of both the stakeholders they represent and the athletes they coach (e.g., Houlihan & Zheng, 2013; Sam, 2012). In most cases, this interest relates to performance enhancement, competitive results, and trophies. According to MacIntyre (1985), however, these interests may lead coaches and athletes to adopt a win-at-all-costs attitude that generates shortcuts and cheating. It is feasible to assume, for instance, that the relatively fixed demands placed on national team coaches to satisfy organizations and stakeholders influence them to adopt coaching methods that result in athletes pushing themselves beyond their physical and mental limits (Barker-Ruchti, Barker, Rynne, & Lee, 2016). Research does indeed show that elite athletes’ experiences of high expectations and competitive pressure increase their vulnerability to burnout, poor mental health, and risk-taking behaviours (Gustafsson, Hassmén, Kenttä, & Johansson, 2008; Hughes & Leavey, 2012; Rice et al., 2016; Safai, Baker, & Fraser-Thomas, 2014). Other “workplace” stressors, including public scrutiny through mainstream and social media, group dynamics, especially in team sports, and injuries, increase this athlete vulnerability (Hanton, Fletcher, & Coughlan, 2005; Johnson, 1997).

Given the dilemma between the demands that coaches face and the potential negative consequences these may cause, various scholars have suggested that coaching should focus on not only skill learning and physical aspects of performance enhancement, but also a broader “taking care of” athletes (Hardman, Jones, & Jones, 2010; Jones & Turner, 2006). Such coaching is commonly referred to as holistic, and being:

*athlete-centred*, and focused on enhancing the self-awareness, and growth and development (across three domains of learning) of the participant … Athletes are expected to analyse, think and make important decisions. (Lombardo, cited in Cassidy, 2010, p. 441, emphasis in original)

Several scholars have linked athlete-centred coaching to “care(ful) actions” that include giving time, being there, engaging in dialogue, showing sensitivity, and empowering athletes (Jones, 2009; Jones, Bailey, & Santos, 2013). In contrast to what Lawson (2005) proclaims, we believe that such actions have the potential to take care of athletes’ health and well-being, and to create and instil in athletes a good balance between performance enhancement, pressure to perform, and health and well-being (e.g., Annerstedt & Lindgren, 2014; Barker-Ruchti & Barker, 2015; Barker-Ruchti, Barker, & Annerstedt, 2014; Hardman & Jones, 2011).

Care(ful) coaching is not only about caring coaching practices; according to Hardman et al. (2010), it also demands of coaches that they model good moral character. Such virtue (Hoveid & Finne, 2014; Jones et al., 2013) is argued to respond to ethical and moral questions of acting “well” and getting it “right” (Jones, 2017). A number of sport coaching and education scholars discuss coaches’ virtue of care (e.g., Hoveid & Finne, 2014; Jones, 2017; Jones et al., 2013). In this literature, caring is seen as an outcome of virtues that reflects a genuine concern for and ethical treatment of others. In specifically focusing on elite sport, scholars find that the elite coaches featured in their research demonstrate genuine caring, which they enact through interest in the athletes’ lives outside sport and inclusive training environments (Annerstedt & Lindgren, 2014). Furthermore, scholars find that the elite coaches’ careful actions include respecting, valuing, involving, engaging in dialogue with, listening to, and supporting players, as well as treating them as human beings, and giving them the confidence and feelings of responsibility to try (e.g., Annerstedt & Lindgren, 2014; Barker-Ruchti et al., 2014; Jones, 2009). Careful coaching, however, may be challenging if the sporting climate is results based (Purdy, Potrac, & Paulauskas, 2016) and to date, little research has focused on examples of, and the ways in which coaches implement coaching methods that develop athletes holistically and successfully. Thus, in this article, we aim to explore how an exclusive sample of women’s national football team coaches described how they implement careful coaching while facing social and organizational pressure to win medals. Specifically, we answer the following research questions: (1) How do the women coaches approach coaching, or in other words, how do they understand their role as coaches? and (2) What coaching strategies do the women coaches implement to fulfil their coaching approach?

### Theoretical framework

To explore how women coaches describe how they implement careful coaching while facing social and organizational pressure to win medals, we drew on Noddings’(2003, 2012a, 2012b) ethics of care. We chose this author’s conception for two reasons. First, Noddings’ ethics of care prioritizes the care process over results-based outcomes (i.e., school grades and competition results). Secondly, sport coaching scholars (e.g., Jones, 2009; Purdy et al., 2016) have argued that coaches who adopt care(ful) actions are able to ensure the well-being and ethical development of their athletes despite being under pressure to produce results-based outcomes (Jones, 2009).

For Noddings (2003), caring is based on relationality and dependent on interactions between the carer and the cared-for. She outlines that the carer, the recipient of this care, and the recognition of the recipient that he or she is being cared for, are the three key elements that define a caring relationship. Gordon, Benner, and Noddings (1996) argue that such caring relationships foster recognition, realization, growth, development of protection, empowerment, culture, and possibility. In a coaching context, such caring would mean that the coach embraces the role of the one who cares and the players being cared for (Purdy et al., 2016). Caring would mean that the coach “does” caring, in that he or she tries to see, hear, or feel what his or her athletes are trying to convey. Noddings (2002) defines such actions as “engrossment”. At the same time, the coach would need to focus on the particular needs, wants, and interests of the cared-for athlete, which means that the carers (i.e., coaches) need to put aside their own interests. Noddings (2002) calls this emphasis “motivational displacement”.

Noddings (1984) further writes that ethics of care aims to influence people to engage in caring relations. The modelling, dialogue, practice, and confirmation of caring and caring relations are key to achieving such a pedagogical climate. Modelling refers to when the carer demonstrates to others what caring means and what actions it entails (Noddings, 1995). Dialogue refers to talking, listening, sharing, and responding in order to understand, empathize, and appreciate, which allows the carer to shift the focus of the interaction to the needs of the cared-for (Noddings, 1992). Practice refers to how the attitudes and ways of thinking are shaped. Lastly, confirmation refers to an act of affirming and encouraging the best in others in order to develop positive relationships, which is a result of having established trust (Noddings, 1992). In the context of elite sport, Noddings’ (cf. 2012a, 2012b) ethics of care means that coaches must accept moral role-modelling. In so doing, coaches will be in a position to consider, and engage with, the various social and moral issues (e.g., anger, envy, fear, inauthentic behaviour, and cheating) that they will encounter as part of their professional practice (cf. Noddings, 2012b).

## Methods

This study follows the qualitative methodology of interpretive description (Thorne, Reimer Kirkham, & O’Flynn-Magee, 2004). Interpretive description acknowledges the constructed and contextual nature of human experience and allows for shared realities (Thorne, 2008). The design strategies in interpretive description follow grounded theory, naturalistic inquiry, and ethnographic principles (Thorne et al., 2004), which represent scientific approaches that are open ended and explorative, and generate categories that describe phenomena contained in gathered data. To capture the women coaches’ complex and shared realities of negotiating results-based expectations and the holistic development of athletes, this study relied on in-depth interviews to produce empirical data. The design in this study is useful for our intention to understand how a sample of Swedish women’s national football coaches unanimously described how they carefully handle the challenges of producing competitive results while at the same time ensuring the holistic development of their athletes. The authors of this study are experienced in qualitative research methods and have solid knowledge of the Swedish sport system and sport coaching literature, specifically with regard to athlete-centred and holistic coaching, sustainability in elite sport, and athletes’ health and well-being.

### Research procedure

The data presented in this article stem from an international project that examined the career pathways of top-level women football coaches (Barker-Ruchti, Lindgren, Hofmann, Sinning, & Shelton, 2014). To recruit these coaches, the sample criterion was a highest level coaching position, which we determined to be senior national, age-specific national team, and assistant or special player positions (e.g., youth team, goalkeeper coaching). Recruitment took place through the international research team identifying relevant women coaches through personal knowledge and internet searches, and contacting national football federations to obtain the identified coaches’ contact details. The federation, and later the coaches, received information about the project, ethical safeguarding, and participation requirements according to the ethical guidelines provided by the scholars’ national ethics commissions (HSFR, 1991). Upon agreeing to participate in the project, the coaches signed a consent form and dates and locations for the interviews were identified. The final international sample consisted of 19 top-level women football coaches.

### Data production

Data production involved an in-depth interview (Rapley, 2004) following life-history principles (Wolcott, 1994). Life-history interviews are very open ended, allowing research participants to retrospectively recount their lives or life phases. A comprehensive interview schedule, which was based on a constructivist view of learning, was developed to guide the research team in conducting the interviews. Interview questions were themed, starting with: (1) entrance into football; (2) coaching career; (3) social learning; (4) contacts with relevant others; and (5) coaching-specific aspects. While following the first four interview themes, the interviewees were given as much freedom as possible to recount their sport, coaching career, and coaching experiences. In the fifth theme, the women coaches were asked to speak about their coaching knowledge, coaching experiences, and coaching philosophy.

Each interview took around 90–180 min (140 min on average). All interviews were audio-recorded, transcribed professionally, and checked for accuracy by the authors. Where possible, the research team members interviewed the study participants in the coaches’ mother tongue; otherwise, the English language was used. Where necessary, the transcripts were translated into English.

### Data analysis

Data analysis began with the aim of understanding the coaches’ career development. During this process, the two authors of this article recognized that the Swedish coaches spoke of coaching as a caring activity, a description that differed from the bulk of coaching literature that demonstrates authoritarian and controlling coaching, yet followed an emerging body of research presenting evidence of caring, holistic, and athlete-centred coaching (Annerstedt & Lindgren, 2014; Barker-Ruchti et al., 2014). Data analysis thus turned towards also exploring the coaches’ statements relating to careful coaching. For this analytic procedure, we adopted an abductive data-analysis approach, which allowed us to engage in a dialectic process of considering data and drawing on theory. Abduction means avoiding theoretical imposition on the one hand, and acknowledging that theoretical interpretation can strengthen empirically based conclusions on the other hand (Dubois & Gadde, 2002). As we are both familiar with caring/careful, holistic, and athlete-centred coaching literature, we responded to the need for theory by more specifically adopting Noddings’(2003, 2012a, 2012b) ethics of care. Thus, the theory was not contemplated and used at the outset of the analytic process, but adopted as data indicated caring coaching. Thus, the study was explorative (Dubois & Gadde, 2002) and interpretive descriptive (Thorne et al., 2004) in nature.

The data-analysis procedure specific to the five Swedish women coaches followed five steps. While this analytic procedure may look like a linear process, it actually involve going back and forth between decontextualization and contextualization, which, although complex, creates analytic depth. First, the first author read each interview transcript a number of times as open-mindedly as possible to obtain an overall impression of the coaches’ interactions with players regarding their philosophy, knowledge, and experiences. Secondly, meaning units that responded to the aim of the study were coded into keywords or key phrases. An example of a meaning unit we gave was: “I try to help them to have patience. They believe that one must be in the best environment when you are 15 … and I know that they need support to choose wisely”. The key phrase highlighted from this meaning unit was “supporting patience behaviour”. Thirdly, we moved between the empirical data and the literature to identify adequate theories, as mentioned before, which would help us to acquire a deeper understanding and better interpretation of the coaches’ handling of their players.

Fourthly, the first author compared the key phrases to each other to find similarities and differences, which were then sorted and arranged into seven tentative categories: (1) to handle elite players’ challenging behaviour; (2) to promote fair play; (3) to promote elite players’ development; (4) to see every player as a human being; (5) to listen to and take care of the players’ needs; (6) not to risk players’ health; and (7) to have a good dialogue with the players. Fifthly, the two authors discussed, compared, and contrasted the seven categories in relation to the concept of “caring”. It was in this step that the seven tentative categories were combined into the three coaching strategy categories of (a) promoting development, well-being, and sustainable elite performance; (b) listening to the players’ voices and having dialogue; and (c) creating a positive environment and promoting fair play (research question 2). No data related to the aim of the study were excluded as a result of a lack of a suitable category or fitting into more than one category. The underlying meaning of the three categories was formulated into the theme of “Holistic perspective of every person and managing performance expectations”. This theme reflects research question 1 and thus the coaches’ approach to coaching. This overarching theme represents the thread of the underlying meaning (i.e., latent content), through the meaning units, keywords, or key phrases and the categories on an interpretative level (Krippendorff, 2013). Before presenting these results, we wish to present the five Swedish women coaches and illustrate the Swedish football context in which they participated at the time of the interview.

### The Swedish football context

At the time of our study, the five women coaches held five of a total of seven possible women’s national football team coaching positions (i.e., women’s senior national team and youth team positions). Table I presents the sampled coaches, who are each named with a pseudonym.

**Table 1.** Coaches’ positions, education and football playing career (*N* = 5).

	*n*
**Coaching position**	
National coach of the Swedish A-level national football team	1
Assistant coach of the Swedish A-level national football team	1
National coach of the Swedish youth national football teams	3
**Education**	
University education qualifications	4
Highest level Swedish football education	4
**Football playing career**	
Highest national and international levels as senior player	1
Highest national level as senior or junior player	3
Lower national level as senior player	1

In addition to the characteristics presented in Table I, the sampled coaches participated in a football context particular to Sweden. In this country, football is one of the most popular sports for women and girls (Swedish Sports Confederation, 2015). Currently, women football players are successful competitively, with the Swedish women’s national senior team having been one of the best worldwide since the 1970s (Hjelm & Olofsson, 2003). This success rate reflects organizational advances which, since the middle of the 1970s, have entailed a system to educate women football coaches (Hjelm & Olofsson, 2003). This education programme has shown success in that, since 1973, three women have held the position of women’s senior national football team coach (of a total of 10 positions over this period) (Nilsson & Börjesson, 2014). Women have also held a number of the youth national football team coach positions.

## Results and discussion

### A holistic perspective of every person and managing performance expectations

The overarching theme, “Holistic perspective of every person and managing performance expectations”, represents the women’s approach to coaching (as per research question 1). The coaches unanimously agreed that they see their players as both players and humans, and their coaching decisions as based on moral insight and caring. The coaches described that it is important to them to give players a sense of being present, being willing to listen, and conveying interest in the players. According to Noddings (2002), this open and non-selective perspective can be regarded as engrossment and a motivational shift (i.e., motivational displacement) towards the players (Noddings, 1995). Indeed, the women described that they aim to influence the players’ development, moral character, health, and well-being. This finding is in line with other scholars who suggest that holistic coaching develops the whole person (Jones & Turner, 2006; Lawson, 2005). From Noddings’ (1992, 2002) point of view, it is further possible to say that the women coaches’ single act of caring for players provides confirmation of the players’ selves. However, to confirm selves and encourage development, coaches must know the players individually so that they can recognize what their desires and needs are. For the coaches in this study, they did not meet their players on a regular basis because players also participated in club teams. To overcome this limited contact, the coaches described that they make extra efforts to develop and sustain positive interactions and trusting relationships with players, strategies that can be seen as confirmation. The coaches also spoke of how they engage in true dialogue with the players, which Noddings (2002, p. 287) defines as “mutual exploration, a search for meaning, or the solution of some problem”. Building on this holistic approach to coaching, the women coaches adopted a number of coaching strategies (research question 2), which we will illustrate below. Before doing this, however, it is important for us to demonstrate that the coaches outlined that they face pressures to produce performance results, a context that some scholars have argued prevents sustainable (read holistic, caring) coaching practices (e.g., Lawson, 2005).

In the case of the Swedish women coaches, however, the above results demonstrate a holistic approach to coaching, despite performance result expectations. This negotiation was possible because the coaches clearly understood their context and role, and the expectations placed on them. Mary, for instance, stated: “… My main job is to prepare and win the next game. That is how it is”. In a similar way, Carolyn said, “When you step up to the highest level in the national team, then it’s results … then it’s more like life or death”. In this statement, Carolyn refers to both the security of her coaching position and her players’ change in play and qualifying for or remaining on national teams. Thus, through their coaching they must influence player-performance to satisfy the results-based demands to ensure the players’ playing and coaches’ job security. Such security is, however, only possible if players and coaches are healthy (i.e., uninjured) and well. Given that the coaches spoke of the high demands they face, and that they have limited time to get to know the players, they need to plan their activities well in order to achieve “good” and sustainable national team activities. Susan said:

The step from being responsible for a club team to being in charge of a national team is huge. Here you are alone … It requires more preparation. I get these players that I don’t know that much, and I don’t meet very often … Preparations have become even more important. I have gone more on gut feeling before [in club teams] … but I have realized that it is not possible to go on gut feeling, it’s so short-sighted and result-driven. Other concerns that I have had are: how do I make it as good as possible, how do I get the players to understand the policy we have and what we accept and don’t accept?

Susan’s statement reinforces that national team positions are different from club-based positions. National team coaching positions are about greater pressure, greater responsibility, and more difficult working conditions because coaches do not see their players on a regular basis and thus they have fewer opportunities to get to know the players. Lastly, national team positions are less secure for both players and coaches.

The first category, “Promoting development and well-being”, contextualizes how the women coaches are faced with results-based expectations, yet, despite these pressures, focus on the holistic development of players, and their health and well-being. In this regard, the coaches’ knowledge of holistic player development is significant. The second category, “Listening to the players’ voices and having dialogue”, includes creating a dialogue with and listening to the players (e.g., Annerstedt & Lindgren, 2014; Barker-Ruchti et al., 2014; Jones, 2009). The third category, “Creating a positive environment and promoting fair play”, is linked to the theme because positive environments are significant in getting the athletes to continue their efforts in elite sport (Fahlström, Gerrevall, Glemne, & Linnér, 2015) and moral education is important to prevent players from adopting risky behaviours.

### Promoting development, well-being, and sustainable elite performance

The women’s national team coaches recognized how important it is to foster their players to become senior players, while at the same time allowing them room to develop as players. The youth national coaches spoke of how they struggle to ensure athletic development while maintaining player health and well-being. One specific strategy mentioned to overcome this struggle was educating youth players and parents about overtraining. They described how they provide advice on gradual athletic progression, and injury and burnout prevention. Theresa’s statement illustrates the importance of the support she provides to players:

I try to help them to be patient. They believe that one must be in the best environment when you are 15 … then it is in the local newspaper and you get attention in one way or another. But unfortunately, that doesn’t indicate so many good parts in terms of development for them. … when you are 23 or the average age of the national team is right now 27.5 or something like that, and you’re 15 now, in about 12 years that is when you should be at your best. Which I believe, and I know that they need support to choose wisely. But the parents shouldn’t make the decision for them, parents often want something else.

How the coaches spoke about their reasoning for education reflects their sensitivity to and caring for the young players, and mirrors what Noddings (2002) says about the interests of the cared-for regarding support, helping them to achieve (long-term) goals, and empowerment, and what this scholar (1992, 2002) calls motivational displacement. The coaches indicated that they see patience and long-term goals, rather than rushing success early in a career, as important (Noddings, 2002).

The coaches said that their personal experience of being young elite players and their professional coaching experiences influenced their standpoint. Theresa said:

Precisely in view of the fact that many of the young players become injured, sooner or later. Why is that? Because you’re not trained like that. But because I was also often damaged, I’ve drawn from my own experiences as what physical therapists and so have told me. It’s different things, but also a genuine interest in humans, how are we going to get as many people as possible to continue playing football?

As Jones (2009) argues, coaches’ caring requires understanding of the athletes they care for. As a rationale for the caring and sustainable strategies, Theresa’s statement, “We have a genuine interest in people and to get as many people as possible to continue”, is representative of the five coaches’ careful actions.

The senior national team coaches also spoke of their goal to promote their players’ social development. In their stories, the coaches outlined that they intend to develop not only technical and tactical skills, but also players’ interactions, and to “make each other good” (Mary) by drawing out the best in each other. The statement illustrates how a coach models caring communication, while all players should help and support each other and thus themselves be involved in a caring practice (Noddings, 1984). The women also mentioned that one strategy they use to socially develop their players is to provide positive feedback that they feel reaches the players’ feelings. Mary explained:

When I give the players feedback, my goal is to reach their feelings and their hearts. Watching the video is a perfect method. They can see what is working well. I reinforce with a lot of praise, specific praise: Oh that’s great, look at what you have achieved. They get applause from teammates.

The coaches felt that by stimulating emotions, they are able to foster their players to reflect on their thoughts of, feelings for, and actions towards others (Noddings, 2003). The coaches also demonstrated care for their players’ well-being. The focus on athletes’ health and well-being is obvious in other studies on national team coaches (e.g., Annerstedt & Lindgren, 2014); however, it contradicts research into football, which demonstrates a lack of concern for athletes’ health and well-being (Brown & Potrac, 2009; Cushion & Jones, 2006). The women also spoke of how their view of the players as whole people influences player well-being. Susan said:

I try to work with the wholeness, we’ll help them feel better … So somewhere I take responsibility for the whole person, although we may think that it is their responsibility and this is football. So I think it is important that we take the responsibility and have the conversation, that you are good enough as a human being.

In this respect, there is a need to engage in an ongoing discussion about the problems that come with the demands that young elite athletes feel, and it is important to support the athletes’ overall development, not only their performance. Focusing more on the athlete as a person is what Hardman et al. (2010) point out is a more holistic, value-based approach to coaching.

### Listening to players’ voices and having dialogue

The coaches expressed that over their coaching careers, their coaching changed from an autocratic to a more democratic style. In so doing, the women described that they have increasingly begun to listen to and include players in the coaching process. The women’s national team coaches described that they now listen more attentively to the players, talk less, and ask more questions. In this regard, Mary said:

I’ve moved on from “I know best” to “there is no way it is like that; others know more”. Clarity and pointing has changed to inviting others in order to understand the ones I coach, and asking questions. As I said, it makes them conscious.

The coaches further expressed that they have developed sensitivity towards young people’s vulnerability within an elite sport culture. This listening to players’ needs and understanding their vulnerability translates into encouraging players to seek meetings with the coaches. This is an example of caring attention, receptivity, and sympathy, which Noddings (1992) called engrossment. Such engrossment represents an honest interest in and engagement with the cared-for (i.e., players), which in turn leads to motivational displacement, or coaches’ selfless actions to respond to players’ needs (Noddings, 1992, 2002). The junior coaches described being particularly fond of this careful action and to provide confirmation to the players by establishing trust (Purdy et al., 2016). Susan stated:

I say [to the players], come to me if there’s something. They should dare to call, dare to knock on my door, and say: Can I talk a little? What I’m working with now is a very remarkable, extraordinary demand on young people. Or not remarkable, but a heavy demand. We’re talking about 15 to 17 years of age where a lot happens, and then they perform at their maximum. Therefore, being the best when it matters the most. I’m supposed to select them, and this is an age when so much is happening and there are very important things. It’s a little absurd, the reality we have here. But to see the whole person, I think that is very important.

What this coach refers to is that a sincere interest in the player as a person, a virtue that ignores self-interests and instead focuses on the young elite player’s needs, is important (Noddings, 1992).

The coaches also spoke of how they hold dialogues with their players before games, during which they discuss expectations and feelings. Theresa explained:

We always have individual dialogues. When we were away, last in [another country], we began with the individual, how they feel and what they are competing for, and what we expect from them. We also have dialogues about what they expect from us and how they can contribute to the mood in the group so we can perform as well as possible during these days. Then at the end of the week, we follow up with another dialogue.

Talking and listening to players also applies during games. Scholars who advocate holistic coaching agree that allowing players the freedom to decide what affects them can increase player motivation (e.g., Martindale, Collins, & Daubney, 2005). Susan explained how she lets her players participate in decisions by asking questions, rather than telling them what to do:

I am a lot about creating security, participation, and responsibility, so I work with a lot of group discussions. During half-time, for example, I will go into this more and talk about how it feels out there. Okay, but in defence, how does it feel? Well, we feel like this and this and this. How do you think we’re going to solve it? When they come up with things, okay let´s do it. I always have one, two, or three things that I’m going to say, but they will come up with something no matter what. … I want an active group and I feel that I have that.

Furthermore, the coaches were unsure about how they should react or behave in some of the situations they encounter. Susan mentioned, for instance, that she and her coaching team discuss and struggle with how to handle players who show non-conforming behaviours when it comes to the accepted standards of being a team player. Susan expressed:

Football is a team game and we do it together, and then you have to subordinate to that piece and to do it with joy … We have had a discussion on how we manage the perhaps more difficult persons and how we work with them. We’re talking about those with diagnoses or cultural difficulties that do not fit into the team. A player could say “I do not play in offence, I’m good here, and this is what I do”. Can you have such a person on the team?

The player’s comment that Susan referred to could easily be interpreted as selfish, and irritate a coach. Susan, however, voiced how although she did not agree with this player’s request, she listened and attempted to create a productive dialogue with the player. This listening and creation of a dialogue can be regarded as a form of caring for the player, to show patience and comprehend her reality (Noddings, 1992), and can also stimulate the players to reflect, which Noddings (2002) regards as a particularly powerful tool to promote and build ethical ideals. Susan explained further:

Because it is a team game, I talk with them about choice. I don’t decide what they should choose. … They only want to play based on their own requirements and ideas, but then I say we have a dialogue. Then we sit in groups … so they have to take that discussion with them and work with it.

We postulate that the dialogue between coach and players allows the two agents to develop shared understanding, empathy, and appreciation, and deeper knowledge of one another (Noddings, 1992).

### Creating a positive environment and promoting fair play

The coaches articulated that they think it is important to create a positive environment for the players through good teamwork with the coaching team. However, being a head coach and being in charge are also challenging, not least because the coaching team members change and the coaching staff have irregular contact with the players. Theresa said:

I think it is important that the [coaching] team works well together, because it spreads to the girls. Given that our team consist of different people travelling with us almost every time, this puts extremely high demands on me.

The coaches said that they aim to create a positive team environment, in which every player is asked to contribute with positive behaviour. This can be regarded as a form of what Noddings (1992) called values practice. This means that coaches provide players with opportunities to collaboratively practise care, from which individuals learn how to care and contribute to a “community” (i.e., Swedish team) in which they participate. However, this is not always easy. The coaches described how, at times, an individual player’s behaviour or cooperation challenges their aim to create a positive environment. In such situations, Mary said that she finds it important for all team members to know that “You’re each other’s environment”. What Mary meant by this statement is that everybody’s actions and behaviours affect the environment. This coach also stated that she feels that coaches and players help each other to achieve their best, and that players working and sticking together to achieve team goals is important. Creating a supportive environment and focusing on problem-solving in active collaboration reflects a health-promoting perspective (WHO, 1986). This relational practice may even foster growth and empowerment (Gordon et al., 1996). The coaches also articulated that they consider a positive environment to involve respect between all players. Indeed, the coaches considered respect as an important starting point in their development of the players’ character and fostering moral virtue and ethics of care. Susan, for instance, said that she reacts immediately if players do not respect one another:

I don’t accept any bad treatment. I feel that in a new group if some girls do an “eye-rolling” thing, I will comment on it directly. After all, I think I need to be clear about it, and I think this is important. If we are a team, then there are pieces that are important. The conversation is important so they be aware of [what’s happening]. I have mentioned this before; individual conversation is important.

In football, fair play is a fundamental part of the game and represents the positive benefits of playing by the rules and respecting others (FIFA, 2016). The coaches expressed that they are morally obliged to behave according to their idea of “good” practice. They assumed that this strengthens their influence on the players. In other words, the coaches do not merely tell the players to care; they demonstrate their caring in their relations with the players, which can be regarded as modelling (Noddings, 1995). The coaches also mentioned that they act in relation to humanistic and fair-play values, mainly in order not to risk the players’ health and well-being. This coaching approach is an example of moral character virtues, representing good discipline and professionalism (Hardman et al., 2010). Thereby, the coaches saw themselves as providing their players with opportunities to cultivate moral character. Mary expressed:

I’m very proud of women’s soccer and what it stands for. I myself stand and say that this is “fair play”. I have a story of a job I had: the goalkeeping coach stepped forward and said to the girls that if it’s going to hell, you take a free kick (i.e., intentionally fouling a player to prevent a goal from being scored). Then I stepped up and apologized for him, that we had not talked about this. I expect everyone here to adhere to my philosophy. This is an important thing and I decide on values, you could say. … we are not taking any free kicks because we’re better than that. For if something is free and we’re “taking” a free kick and she gets injured, then I can truly go back and say that I don’t like it and then I cannot do this. We play tough but we take no free kicks.

Mary’s statement is not an isolated quote about fair play and moral standards; the women coaches clearly outlined that they stand for fair play in general and do not want to risk players’ health. Mary’s integrity exemplified an ability to think and act in relation to particular values (e.g., working hard but fair) and caring at the right time, for the right reasons, and with the right feelings (Standal & Hemmestad, 2011). In other words, Mary’s perspective serves as an example of taking on responsibility to foster player morals, and can be interpreted as modelling, where the coaches show in their own behaviour what it means to care (e.g., Noddings, 2012a, 2012b). As Noddings (2002, p. 287) wrote, modelling may be especially powerful “because its very authenticity is morally significant”.

All of the women’s national team coaches had long and diverse experiences of being a football player on a national or international level and as highest level coaches. However, when it comes to acting for the greater good, in general, the coaches highlighted the importance of what they have learnt from their families or significant others about knowing right from wrong, fair from unfair, and caring. Mary said:

I grew up in a family where we talked about solidarity, where one should help the weak. Of course, all of this is so much more than just soccer. I therefore believe that the passion for soccer has taken me over all opposition. … Another crucial thing has been to not take myself so seriously.

Mary’s statement is one example of where it is possible to associate her clear morals with growing up in a country that is shaped by democratic principles (Enoksen et al., 2014), and which has created a sport-for-all movement, a particular national sport policy, and a focus on athletes’ education and well-being (Barker-Ruchti et al., in press; Ronglan, 2015). Furthermore, Mary’s quote of not taking herself too seriously exemplifies how she realizes that she can learn from others, which is indeed something that the women coaches mentioned.

## Conclusion

The aim of this article was to explore how an exclusive sample of women’s national football team coaches described how they implement careful coaching while facing social and organizational pressure to win medals. In particular, we aimed to consider the coaching approach that the coaches adopt and the coaching strategies that they implement to fulfil this approach. Our findings demonstrate that the five coaches unanimously adopted a holistic approach to coaching. This approach extended performance enhancement to include the coaching strategies of listening to the players’ voices, engaging in dialogue and meeting the players’ needs, and focusing on players’ health, well-being, and sustainable elite performance. Using Noddings’ ethics of care, the findings demonstrate engrossment in that the women coaches try to see, hear, and feel what the players are trying to convey; and bracket off their self-interests (i.e., motivational displacement). The findings also demonstrate that the coaches provide confirmation to the players through building trust as well as encouraging players’ moral development. However, our data demonstrate that because the women did not regularly see their players at the national team level, they were limited in establishing opportunities for the players to collaboratively practise care and contribute to their national team community.

At present, although calls for caring, holistic, and athlete-centred coaching are common, only a few empirical examples exist of how elite or national team coaches manage the negotiation of results-based expectations and holistic athlete development (exceptions are Annerstedt & Lindgren, 2014; Barker-Ruchti et al., 2014). Thus, our study of Swedish women’s national team football coaches provides an example of elite-level coaches adopting caring, holistic, and athlete-centred coaching (Hardman et al., 2010; Jones & Turner, 2006), while working at the highest level of competitive sport, and in the case of our women coaches, achieving competitive success. In today’s climate of performance-based results and abusive coaching practices, we consider it imperative to demonstrate such positive cases and holistic coaching strategies. We hope that other scholars see merit in adopting a positive research approach to discover and explore how elite-level coaches negotiate result-based expectations, sustainably develop athletes, and produce top-level athletic results.

### Methodological considerations

To ensure research credibility, we adopted a number of strategies, such as aiming to include all women’s national football team coaches in Sweden to participate in the study, which we achieved, and following a strict analytic procedure. As for methodological limitations, our findings are based on a small and exclusive sample of coaches. Therefore, the findings can only be transferred to similar groups of coaches. Future studies about caring coaching in national football team situations should include male coaches, which could enrich our understanding of ethics of care and potentially add a gender perspective. Another possible limitation of the study was that the interviews focused on several aspects. It is important to note that had specific questions been asked about careful coaching, the women coaches’ responses may have included additional aspects of how they manage the players carefully and holistically.
